# Electrochemical Sensing of Dopamine Using Polypyrrole/Molybdenum Oxide Bilayer-Modified ITO Electrode

**DOI:** 10.3390/bios13060578

**Published:** 2023-05-26

**Authors:** Nadiyah Alahmadi, Waleed Ahmed El-Said

**Affiliations:** 1 Department of Chemistry, College of Science, University of Jeddah, Jeddah 21959, Saudi Arabia; 2 Chemistry Department, Faculty of Science, Assiut University, Assiut 71516, Egypt

**Keywords:** conducting polymer, molybdenum oxide, dopamine neurotransmitter, metal oxide nanoparticles, electrochemical sensor

## Abstract

The electrochemical sensing of biomarkers has attracted more and more attention due to the advantages of electrochemical biosensors, including their ease of use, excellent accuracy, and small analyte volumes. Thus, the electrochemical sensing of biomarkers has a potential application in early disease diagnosis diagnosis. Dopamine neurotransmitters have a vital role in the transmission of nerve impulses. Here, the fabrication of a polypyrrole/molybdenum dioxide nanoparticle (MoO_3_ NP)-modified ITO electrode based on a hydrothermal technique followed by electrochemical polymerization is reported. Several techniques were used to investigate the developed electrode’s structure, morphology, and physical characteristics, including SEM, FTIR, EDX, N_2_ adsorption, and Raman spectroscopy. The results imply the formation of tiny MoO_3_ NPs with an average diameter of 29.01 nm. The developed electrode was used to determine low concentrations of dopamine neurotransmitters based on cyclic voltammetry and square wave voltammetry techniques. Furthermore, the developed electrode was used for monitoring dopamine in a human serum sample. The LOD for detecting dopamine by using MoO_3_ NPs/ITO electrodes based on the SWV technique was around 2.2 nmol L^−1^.

## 1. Introduction

The real-time detection of biomarkers with high sensitivity and accuracy could play a potential role in the clinical diagnostics of several diseases. Biomarkers include genes/gene products, enzymes, proteins, antigens, cells, hormones, etc. The fast and accurate monitoring of the concentrations of the biomarkers inside the human body, as well as in the human fluids, has great significance in the investigation of several human physiological functions besides diagnosis and disease inhibition [1]. Recently, several biomarkers were used for detecting different types of cancer, neural diseases, metabolic diseases, etc. [2,3,4,5,6,7,8,9].

The dopamine (DA) neurotransmitter is considered to be a biomarker for several diseases, including Parkinson’s disease, Huntington’s disease, and schizophrenia [10,11,12,13]. Several techniques were applied to detect DA, including electrochemical [14,15,16,17,18], optical [19,20,21], spectroscopic [21,22,23], and chromatographical [24,25,26,27,28,29,30] techniques. Although spectroscopic-based techniques have high sensitivity, they are time-consuming and need expensive equipment. The electrochemical techniques were used for monitoring the neurotransmitters due to their advantages, which include their fast response, ease of use, and high sensitivity [14]. The main challenge for developing an electrochemical DA sensor is its capability to detect DA in complicated matrices in the presence of ascorbic acid (AA) and uric acid, which are the main interferences. Therefore, several modified electrodes were reported for detecting DA at very low concentrations in biological samples containing different interferences. Label-based electrochemical sensors of biomarkers are distinguished from other techniques by their high selectivity and sensitivity. On the other hand, label-free electrochemical sensors of biomarkers could solve the main drawbacks of label-based electrochemical sensors, including the high cost and low stability. Hence, several label-free electrochemical sensors were reported to enhance sensitivity and be more cost-effective. 

Nanomaterials were used to modify several electrodes and enhance their conductivity, biocompatibility, stability, and sensitivity [31,32,33,34]. Gold nanoparticle (Au NP)-modified electrodes were also used for enhancing DA sensing [35]. CuO NP- [36], Ag-doped CuO NP- [37], Co_3_O_4_/CuO nanocage- [38], and g-C_3_N_4_/CuO nanocomposite-based [39] sensors were used for monitoring the DA level. Ionic liquid-supported Ni–metal–organic framework (MOF)-modified GCE electrodes were used for the detection of DA with a limit of detection (LOD) of 60 nmol L^−1^ [40]. Furthermore, Ag NPs anchored onto a CuO porous nanobelt-modified ITO electrode were utilized for DA determination and showed a linear range for the detection of DA ranging from 0.04 to 10 μM, with a LOD of 7.0 nM. Furthermore, the practicality of the developed sensor has been evaluated by analyzing DA in human serum samples [37]. An Au-SiO_2_ nanocomposite-modified GCE-based DA biosensor was reported based on the differential pulse voltammetry (DPV) technique. The large surface area and high conductivity of the Au-SiO_2_/GCE resulted in a high electrocatalytic response towards DA. The modified electrode displayed a linear relationship within a range from 10 μM to 500 μM, with a LOD of 1.98 μM [41]. A Fe_2_O_3_ NP-modified microelectrode was used for in vitro and in vivo DA detection based on the fast-scan cyclic voltammetry (CV) technique. The fabricated microelectrode showed a LOD of 8.76 nM. Furthermore, this microelectrode was used for monitoring DA in a freely moving mouse [42]. CuO and NiO NP-modified graphite electrodes were used for the detection of DA in some biological samples [43]. Carbon NPs (CNPs) functionalized with sulfonic groups encapsulated in silica matrix-modified rotating ring disc electrodes (RRDE) were reported for the detection of serotonin and DA at very low concentrations [44].

Conducting polymers are widely utilized for developing several electrochemical sensors because of their higher conductivity, doping and de-doping processes, cost-effectiveness, biocompatibility, and multifunctional features [45,46,47,48,49,50,51,52,53,54,55,56]. A polymer-incorporated nanomaterial could increase the strength of the electrochemical transmission nanocomposites based on metal oxide/CuO [57]. Several polymers, including PEG (polyethylene glycol), PVP (polyvinylpyrrolidone), PVA (poly(vinyl alcohol)), PAA (poly(acrylic acid)), and polyaniline (PANI) [58,59,60,61,62], were used in the fabrication of a biosensor. Polypyrrole (PPy) is potentially one of the most conductive polymers due to its high conductivity and biocompatibility [63]. Metal oxides/polymer composites could induce new features besides the characteristics of the conducting polymer and the metal oxides [64,65,66]. The use of polymer/metal oxide composites for the modification of traditional electrodes could enhance the electrode’s sensitivity, stability, and repeatability. A PPy–mesoporous silica-modified gold electrode was used for the electrochemical monitoring of DA based on CV and square-wave voltammetry (SWV) techniques. A linear range for the determination of the DA concentration over 10 µM to 1.2 mM with a LOD of 2.5 µM was observed [67].

A CuO/PVA-modified GCE-based electrochemical sensor was reported for the detection of DA; it showed high sensitivity with a LOD of 0.017 μM [68]. Molecularly imprinted polymer membranes of graphene oxide and PPy-modified micropipette tip carbon paste electrode have been used for detecting DA within a range from 6.4 × 10^−8^ to 2 × 10^−4^ M, with a LOD of 1 × 10^−8^ M [69]. Furthermore, a smartphone-based electrochemical DA sensor was reported. The poly(3,4-ethylenedioxythiophene)/chitosan/graphene/screen-printed electrode was used to measure DA; it showed high sensitivity with a LOD of 0.29 µM [70]. Another nanocomposite composed of reduced graphene oxide/multi-walled carbon nanotubes/PPy was reported for sensing DA. The use of this nanocomposite showed a fast response and high sensitivity over a range from 25 to 1000 nM, and it had a LOD of 2.3 nM [71]. Moreover, cobalt ferrite NP- and manganese ferrite NP-modified graphite electrodes were reported to simultaneously detect paracetamol and DA, with LODs of 350 nM and 400 nM, respectively [72].

This paper reported on the fabrication of MoO_3_ NPs/ITO electrodes based on the hydrothermal technique. The MoO_3_ NPs/ITO electrode was used for detecting different concentrations of DA based on the CV and SWV techniques. The MoO_3_ NPs/ITO electrode showed a very low LOD of the DA neurotransmitter. Furthermore, the MoO_3_ NPs/ITO electrode was used for monitoring DA in a biological sample.

## 2. Materials and Methods

### 2.1. Materials

Ammonium heptamolybdate (99.5% (NH_4_)_6_Mo_7_O_24_), pyrrole, ethylene glycol (99.5% (CH₂OH)₂), dopamine hydrochloride, AA, human serum, K_2_Fe(CN)_6_, K_3_Fe(CN)_6_, and phosphate-buffered saline (PBS, pH 7.4) were obtained from Sigma-Aldrich, USA. All solutions used in the experiment were prepared using deionized water (DIW). The solvents utilized in this study were of analytical grade.

### 2.2. Fabrication of MoO_3_ NP-Modified ITO Electrodes

A solution of one g of (NH_4_)_6_Mo_7_O_24_ in a mixture of DIW and ethylene glycol (90:10 *v*/*v*) was made by continuous stirring for about 30 min at normal temperature. Then, the solution was transferred into an autoclave that contained ITO substrate (1 cm × 2 cm) and heated in an oven for 36 h at 180 °C. The obtained molybdenum oxide NP-modified ITO electrode was rinsed with DIW and ethanol. Then, it was dried in an oven at a temperature of 80 °C for 6 h.

### 2.3. Fabrication of Polypyrrole/MoO_3_ NP Bilayer-Modified ITO Electrodes

Different thickness layers of PPy were deposited onto the MoO_3_ NP-modified ITO electrode based on the electrochemical polymerization process. An aqueous solution of 100 µmol L^−1^ of the pyrrole containing 0.1 mol L^−1^ HCl was used as a polymerization bath. Then, the MoO_3_ NPs/ITO substrate (1 cm × 2 cm) was immersed in the pyrrole solution (1cm × 2cm). The polymerization was performed based on the CV technique within a potential window from −0.6 V to 1.0 V at a scan rate of 100 mV s^−1^. Different numbers of cycles were applied to control the thickness of the polymer layer. The obtained PPy/molybdenum oxide NP-modified ITO electrode was cleaned in DIW and dried in an oven at 80 °C for 6 h.

### 2.4. Analyses

The FTIR spectrum of the manufactured PPy/MoO_3_ NPs was recorded with a Pye-Unicam Sp-883 spectrophotometer from PerkinElmer (Waltham, MA, USA). Scanning electron microscope (SEM) pictures were acquired (Quanta 250 FEG, FEI, Thermo Fisher Scientific, Hillsboro, OR, USA). The BET measurements were evaluated by nitrogen adsorption using a BELSORP MIN-II analyzer (MicrotracBEL Corp., Osaka, Japan); the measurement was performed at 77 K. The TGA/DTA studies were collected using a Shimadzu DT = TG-50 thermogravimeter, under a N_2_ environment (Shimadzu, Kyoto, Japan) (Shimadzu, Kyoto, Japan). The Raman spectra were recorded by using a Bruker Senterra Raman microscope (Bruker Optics Inc., Germany) with 785 nm excitation, 1200 rulings mm^−1^ holographic grating, and a charge-coupled device (CCD) detector. The accumulation time was 5 s with a power of 50 mW. 

### 2.5. Electrochemical Measurements

A homemade three-electrode electrochemical cell was used for all the electrochemical measurements. The PPy/molybdenum oxide NP-modified ITO electrode (1 cm × 2 cm) was used as the working electrode; the active surface area of the working electrode was 1 cm^2^. Furthermore, a platinum wire was used as the auxiliary electrode, and Ag/AgCl (Metrohm, 3 mol L^−1^ of KCl) was used as the reference electrode. Different concentrations of DA were determined using these three electrodes. CV and SWV were used for detecting DA neurotransmitters in buffer solution as well as in biological samples. All the electrochemical measurements were performed in PBS buffer (pH, 7.4)

## 3. Results

### 3.1. Synthesis and Characterization of PPy/MoO_3_ Bilayer-Modified ITO Electrodes

**Figure 1:**
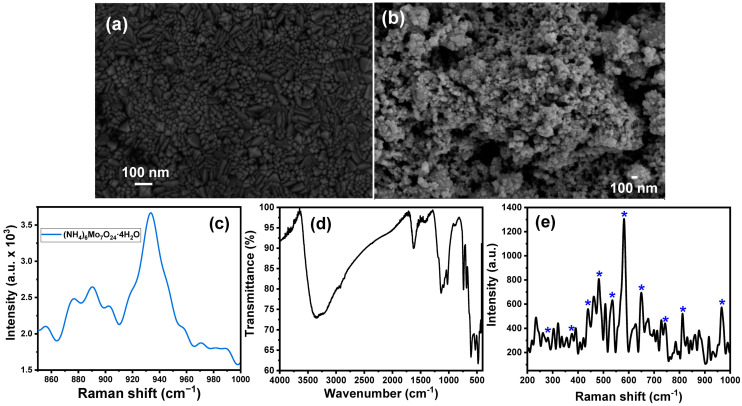
(**a**) The SEM image of the ITO electrode; (**b**) the SEM image of the MoO_3_ NP-modified ITO electrode; (**c**) Raman spectrum of ammonium molybdate; (**d**) FTIR spectrum of the MoO_3_ NPs; and (**e**) Raman spectrum of MoO_3_ NP-modified ITO.

The formation of MoO_3_ NPs and their PPy/MoO_3_ bilayer-modified ITO electrodes were analyzed using different techniques, including FTIR, SEM, EDX, Raman spectroscopy, and N_2_ adsorption. Figure 1a shows the SEM image of the bare ITO electrode and demonstrates the characteristic morphology of the ITO substrate. The morphology of the MoO_3_ NP-modified ITO electrodes was also investigated by the SEM micrographs. The SEM image of the MoO_3_ NPs/ITO electrode shows the formation of tiny nanospheres (Figure 1b). The average particle size of the fabricated MoO_3_ NPs/ITO electrode was calculated using ImageJ (IJ153) software, in which 30 particles were selected and their diameter was studied. The results showed the formation of nanospheres of an average diameter of 29.01 ± 12.47 nm. The distribution of the particle sizes is shown in the supplementary data (Figure S1a,b), which indicates that the particle size range was within the range from 5.59 nm to 54.85 nm.

The Raman spectrum of ammonium molybdate is shown in Figure 1c, which shows a set of peaks at Raman shifts of 856, 876, 890, and 933.5 cm^−1^, which are in good agreement with the previously reported data [73]. Figure 1d illustrates the FTIR spectrum of the prepared MoO_3_ NPs. The FTIR spectrum shows two bands at 1040 and 1110 cm^−1^, which could be attributed to the Mo=O stretching and Mo-O-Mo vibration modes [74,75]. The strong and sharp band at around 1620 cm^−1^ and the broadband centered at 3434 cm^−1^ are attributed to the stretching absorption of the water molecules and the hydroxyl groups [74]. Furthermore, the shoulder peak at 1400 cm^−1^ is related to the Mo−OH bond vibration [75,76]. Moreover, the spectrum shows a band at about 681 cm^−1^ (asymmetric stretching vibrations of the O−Mo−O bonds) and a band at 741 cm^−1^ (stretching vibration of the Mo−O bond) [77,78].

The chemical composition of the MoO_3_ NPs/ITO electrode was also investigated by Raman spectroscopy. Figure 1e displays the Raman spectrum of the MoO_3_ NPs/ITO electrode, which showed many characteristic bands of MoO_3_ NPs. The Raman bands at 580.5 and 742.5 cm^−1^ were assigned to the stretching vibrations of the Mo–O (I) and Mo–O (II) groups, respectively [53]. Furthermore, the Raman band at 279 cm^−1^ O=M=O was assigned to the wagging vibration, while the Raman bands at 650.5 cm^−1^, 812.5 cm^−1^, and 966.5 cm^−1^ corresponded to the stretching vibration of O–M–O, M=O, and M=O, respectively [79,80,81,82].

**Figure 2:**
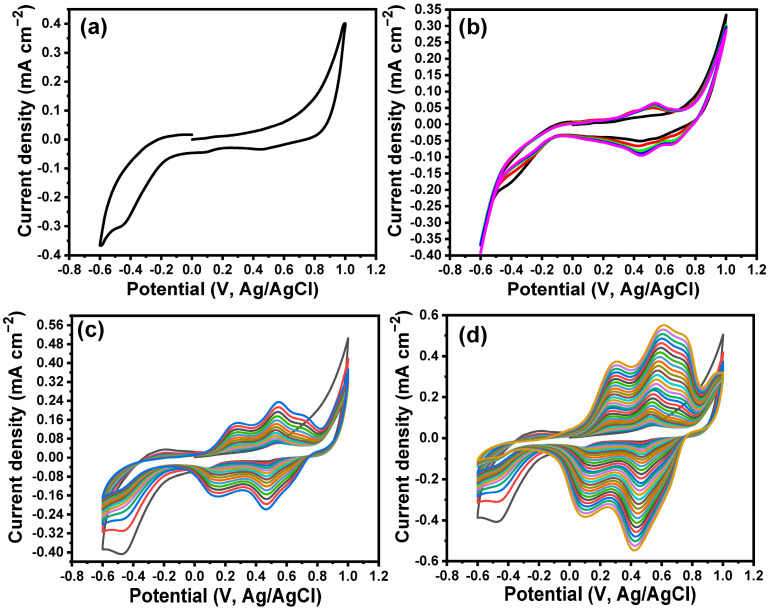
(**a**) CV of MoO_3_ NPs/ITO electrode in 0.1 M HCl in the absence of pyrrole, CV voltammograms of the electrochemical polymerization of pyrrole onto MoO_3_ NPs/ITO electrode at (**b**) 5 cycles, (**c**) 15 cycles, and (**d**) 30 cycles.

A layer of PPy was fabricated onto the MoO_3_ NPs/ITO surface based on the electrochemical polymerization process. The polymerization process was performed based on the CV technique. The thickness of the polymer layer was controlled based on the number of cycles during the polymerization. The CV for the MoO_3_/ITO electrode in 0.1 M HCl in the absence of pyrrole is represented in Figure 2a, which shows a background response without any redox peaks. Figure 2b–d shows the cyclic voltammograms of the electrochemical polymerization of pyrrole after 5, 15, and 30 cycles. The CV response for the first cycle showed redox potential peaks at 0.38 V and 0.43 V. The intensity couples of the redox current peaks were increased with the increasing number of cycles. Moreover, the redox potential peaks were shifted to 0.15 V and 0.27 V. In addition, there was the appearance of new redox potential peaks at 0.47 V and 0.55 V. This behavior is characteristic of the polymerization process. 

**Figure 3:**
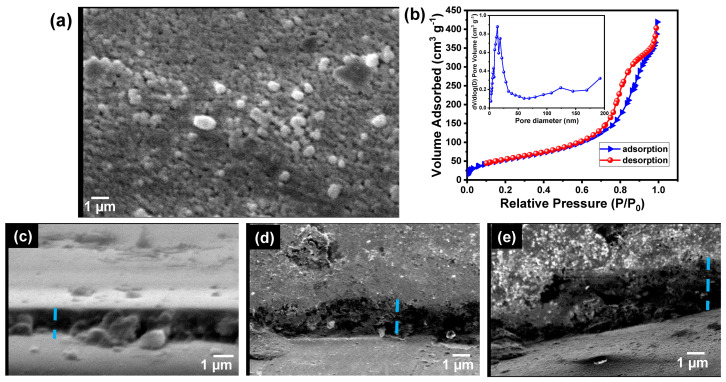
(**a**) SEM image of the top view of PPy/MoO_3_ bilayer-modified ITO; (**b**) N_2_ adsorption/desorption isotherm of PPy/MoO_3_ bilayer; (**c**–**e**) SEM images of the cross-sections of the PPy/MoO_3_ bilayer/ITO electrodes, which formed after different cycle numbers.

Figure 3a shows the SEM image of the PPy/MoO_3_ bilayer-modified ITO electrode. The micrograph shows the formation of larger particles compared with those in the case of the MoO_3_ NP-modified ITO electrode. The average particle size of the fabricated PPy/MoO_3_ bilayer/ITO electrode was also calculated using ImageJ (IJ153) software, in which 30 particles were selected and their diameters were studied. The results showed the formation of nanospheres of an average diameter of 250.69 ± 98.7 nm. The distribution of the particle sizes is shown in the supplementary data, which indicates that the particle size range was within the range from 64.21 nm to 431.10 nm.

The BET method was used to measure the surface area of the synthesized PPy/MoO_3_ bilayer. The formed PPy/MoO_3_ bilayer powder was obtained by scratching the formed layer and using it for analysis. The sample was first heated at 100 °C for 1 h to remove the physio-adsorbed water molecules. Then, the nitrogen adsorption/desorption isotherm against relative pressure was obtained at 77 K. Figure 3b demonstrates the nitrogen adsorption/desorption isotherm, which showed a type IV isotherm (H1) with a significant hysteresis loop. The results indicated the mesoporous trait of the PPy/MoO_3_ nanostructure. The BET surface area, pore volume, and BJH pore diameter were found to be 227.54 m^2^/g, 0.62 cm^3^/g, and 13.91 nm, respectively. The results demonstrated a high surface area compared to the reported polymers and bilayer-based polymers.

The thicknesses of the formed PPy/MoO_3_ bilayer-modified ITO electrodes were investigated by using the SEM. Figure 3c–e shows the cross-section SEM images of the different PPy/MoO_3_ bilayer-modified ITO electrodes, which formed at different numbers of cycles. The results indicate that the PPy/MoO_3_ bilayer’s thickness was increased with increasing cycles. The average thicknesses of the different layers were found to be 0.9, 1.4, and 1.8 µm, corresponding to the polymerization for 5, 15, and 30 cycles. 

**Figure 4:**
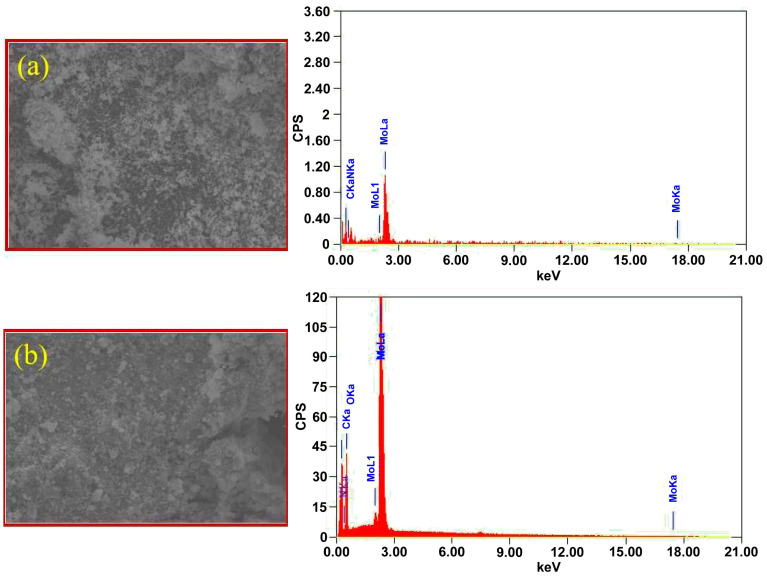
EDX analysis of (**a**) MoO_3_ NPs and (**b**) PPy/MoO_3_ bilayer.

Finally, the EDX was used to quantitatively analyze the elemental composition of the fabricated PPy/MoO_3_ bilayer. Figure 4 shows the EDX of the MoO_3_ NPs and PPy/MoO_3_ bilayer and confirms that the as-prepared material consists of MoO_3_ together with C, N, and O only. Thus, the formed material did not contain any other materials.

### 3.2. Electrochemical Detection of DA, Durability, and Reproducibility of the Fabricated Sensor

**Figure 5:**
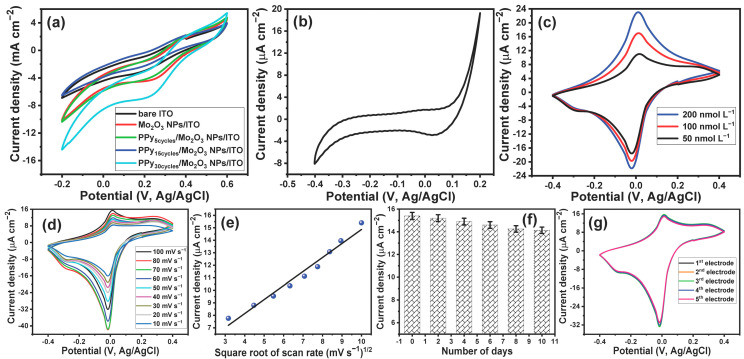
(**a**) CV voltammograms of different electrodes in the presence of 5 mmol L^−1^ of [Fe(CN)_6_]^3−/4−^ electrolyte, scan rate 100 mV/s vs. Ag/AgCl; (**b**) CV voltammogram of PPy/MoO_3_ bilayer/ITO electrode in presence of PBS (pH 7.4), scan rate 100 mV/s vs. Ag/AgCl; (**c**) CV voltammograms of PPy/MoO_3_ bilayer/ITO electrode in presence of different concentrations of DA in PBS (pH 7.4), scan rate 100 mV/s vs. Ag/AgCl; (**d**) CV voltammograms of PPy/MoO_3_ bilayer/ITO electrode in presence of 100 nmol L^−1^ of DA at different scan rates from 10 mVs^−1^ to 100 mVs^−1^; (**e**) the relationship between the square root of the scan rate and the oxidation current peak; (**f**) the durability of the fabricated sensor; and (**g**) the reproducibility of the fabricated sensor.

In the beginning, the effect of the MoO_3_ NPs and the PPy/MoO_3_ bilayer layer of different thicknesses on the conductivity of the ITO electrode was investigated. The electrochemical conductivity of all the modified electrodes was investigated in the presence of 5 mmol L^−1^ of [Fe(CN)_6_]^3−/4−^ in PBS (pH 7.4) and their electrochemical responses were compared to that of the naked ITO electrode. Figure 5a (black curve) demonstrates the CV response of the bare ITO electrode in the presence of 5 mmol L^−1^ of [Fe(CN)_6_]^3−/4−^; it shows a reduction peak at 0.186 V and an oxidation peak at 0.386 V. These redox potential peaks were shifted to become more positive upon the modification of the ITO electrode with MoO_3_ NPs, as shown in Figure 5a (red curve). The CV behavior of the 5 mmol L^−1^ of [Fe(CN)_6_]^3−/4−^ at the MoO_3_ NPs/ITO electrode demonstrated the appearance of a reduction potential peak at 0.25 V and an anodic potential peak at 0.39 V. It is noteworthy that the intensities of the redox current peaks were increased after modification of the ITO electrode with MoO_3_ NPs. Furthermore, the difference between the oxidation and reduction potential peaks was decreased upon the modification of the bare ITO electrode, which indicated the enhancement of the electron transfer rate at the modified electrode compared with the bare ITO electrode. Moreover, the deposition of a 0.9 µm layer of PPy onto the MoO_3_ NPs/ITO electrode showed almost the same current redox potential peaks as those of the MoO_3_/ITO electrode, but it resulted in the enhancement of the electrical conductivity of the MoO_3_ NPs/ITO electrode by about 23% (Figure 5a (green curve)). On the other hand, increasing the thickness of the PPy layer to about 1.4 µm resulted in more enhancement of the electrical conductivity of the modified electrode (Figure 5a (blue curve)), while the deposition of a PPy layer of a thickness of about 1.8 µm showed the same redox potential peaks but reduced the intensity of the redox current peaks (Figure 5a (cyan curve)). The above results confirmed that the modification of the bare ITO electrode with the PPy/MoO_3_ bilayer resulted in the enhancement of the conductivity and revisability of the electrode. Furthermore, the highest conductivity corresponded to the modified electrode with a 1.4 µm PPy/MoO_3_ bilayer; thus, this modified electrode was utilized for all the electrochemical measurements.

The PPy/MoO_3_ bilayer-modified ITO electrode was used for determining DA neurotransmitters dissolved in PBS buffer (pH, 7.4). Figure 5b illustrates the CV response of the PPy/MoO_3_ bilayer/ITO electrode in PBS (pH, 7.4); it shows a background voltammogram in which no redox peaks can be observed. Furthermore, the CV response of the PPy/MoO_3_ bilayer-modified ITO electrode towards the three different concentrations of DA (50, 100, and 200 nmol L^−1^) is represented in Figure 5c. The voltammograms showed a revisable redox behavior with a cathodic peak at −0.02 V and an anodic peak at 0.01 V. These results indicated the capability of the PPy/MoO_3_ bilayer-modified ITO electrode to detect low concentrations of DA neurotransmitters. The effect of scan rate on the electrochemical response of the DA neurotransmitters at the PPy/MoO_3_ bilayer/ITO electrode was studied. Figure 5d shows the cyclic voltammograms of 100 nmol L^−1^ of DA at the PPy/MoO_3_ bilayer-modified ITO electrode under different scan rates from 10 mVs^−1^ to 100 mVs^−1^. The voltammograms showed an increase in the oxidation current peak with the increase in the scan rate. Figure 5e shows the relationship between the square root of the scan rate and the intensity of the oxidation current peaks; the relationship is demonstrated by a linear curve over the scan rate range from 10 to 100 mVs^−1^. The direct proportion between the square root of the scan rate and the intensity of the oxidation current peaks indicated that the oxidation of DA was diffusion controlled. The durability of the fabricated sensor over 10 days was investigated (Figure 5f). The results demonstrated that the prepared sensors had over 91% of their efficiency after 10 days of use. The reproducibility of the fabricated sensor was evaluated by comparing the responses of five electrodes toward 100 nmol L^−1^ of DA (Figure 5g). The responses indicated that the fabricated sensors had almost the same electrochemical conductivity, with a slight variation.

### 3.3. Sensitivity, Specificity of the Electrochemical DA Sensor, and the Interference Effects

**Figure 6:**
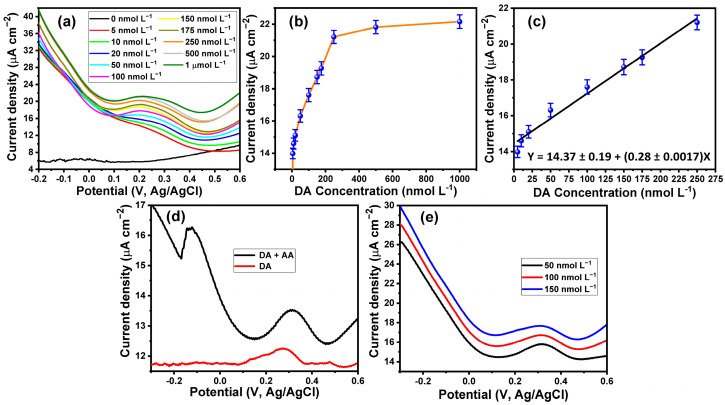
(**a**) SWV voltammograms of different concentrations of DA within a range from 5 nmol L^−1^ to 1000 nmol L^−1^; (**b**,**c**) calibration plot between the concentrations of DA and the oxidation current peak; (**d**) SWV voltammogram of a mixture of AA and DA; and (**e**) SWV voltammograms of different concentrations of DA in human serum.

The sensitivity of the developed electrode toward the detection of DA was tested based on the SWV technique. Figure 6a shows the SWV voltammograms of a wide range of DA concentrations from 5 nmol L^−1^ to 1 µmol L^−1^. The SWVs showed an oxidation potential peak at about 0.27 V. Furthermore, Figure 6b shows the relationship between the DA concentration and the corresponding oxidation current peak. The results indicated that the oxidation current peak was increased with the increase of the concentration of DA until the concentration of 250 nmol L^−1^. Then, the curve reached saturation at concentrations over 250 nmol L^−1^, with a small increase in the current peak with a large increase in DA concentration. The linear relationship between the DA concentration and the oxidation current peak could be observed within the range from 5 nmol L^−1^ to 250 nmol L^−1^ (Figure 6c). The LOD of the developed electrode was calculated according to LOD = 3.3SteDev/slope. The LOD of the DA at the PPy/MoO_3_ bilayer-modified ITO electrode based on the SWV technique was found to be about 2.2 nmol L^−1^. The limit of quantification (LOQ) was also calculated as LOQ = 10SteDev/slope, and it was found to be 6.66 nmol L^−1^. The LOD of the fabricated sensor was lower than some of the previously reported sensors, as shown in Table 1. 

Ascorbic acid is one of the interferences that are present in the blood, and it is oxidized at the same potential as the DA at most traditional electrodes [83]. Figure 6d shows the SWV voltammogram of a mixture of 10 µmol L^−1^ of AA and 50 nmol L^−1^ DA compared with that of DA alone. The voltammogram of the AA and DA mixture showed an oxidation potential peak at 0.31 V corresponding to the oxidation of DA and another oxidation potential peak at −0.13 V attributed to the oxidation of AA. Thus, the developed sensor could detect DA in the presence of other interferences. Moreover, three DA concentrations (20, 50, and 100 nmol L^−1^) in human serum were detected, as shown in Figure 6e. The SWV voltammogram of DA in human serum indicated that the oxidation potential peak was shifted to a more positive potential, at about 0.32 V. The recovery of the 20, 50, and 100 nmol L^−1^ DA in human serum was found at 101.5 ± 0.4, 100.6 ± 0.6, and 99.6 ± 0.4, respectively.

## 4. Conclusions

The PPy/MoO_3_ bilayer-modified ITO electrode was synthesized based on a two-step method, which involved the deposition of MoO_3_ NPs based on the hydrothermal technique and the formation of the polymer layer using the electrochemical polymerization technique. FTIR, SEM, EDX, Raman spectroscopy, and N_2_ adsorption–desorption measurements were used to fully characterize the fabricated electrode. The modified electrode was utilized to determine very low concentrations of DA in PBS as well as in the presence of other interferences such as AA and human serum. The modified electrode had a LOD of the DA based on the SWV technique of about 2.2 nmol L^−1^. Although the fabricated sensor was fabricated based on cheap materials, it had a lower LOD than some of the recently reported DA sensors. Our results confirmed the capability of the modified electrode to detect very low concentrations of DA in human serum; thus, it is a potential sensor for detecting DA in real samples.

## Figures and Tables

**Table 1:** A comparison of the performance of some previously reported DA sensors.

Modified Electrode	Method	LOD	Reference
Screen-printed electrode	DPV	0.29 μmol L^−1^	[68]
GO/MWCNTs/PPy	Amperometry	2.3 nmol L^−1^	[69]
Ag/CuO PNBs	CV	7 nmol L^−1^	[37]
Au—SiO_2_	CV	1.98 μmol L^−1^	[40]
Fe_2_O_3_-modified microelectrode	Fast-scan CV	8.76 nmol L^−1^	[41]
PVP-GR/GCE	CV	2 nmol L^−1^	[40]
Ppy/MoO_3_/ITO	SWV	2.2 nmol L^−1^	Present work

## Data Availability

The data presented in this study are available on request from the corresponding author.

## Supplementary Materials

The following supporting information can be downloaded at: https://www.mdpi.com/article/10.3390/bios13060578/s1, Figure S1: Distribution of the particle sizes of (a) MoO_3_ NPs/ITO, and (b) PPy/MoO_3_ NPs/ITO.
