# Supplementary material for: Electrochemical Sensing of Dopamine Using Polypyrrole/Molybdenum Oxide Bilayer-Modified ITO Electrode
Source: Biosensors (Basel). 2023 May 26;13(6):578. doi: 10.3390/bios13060578 (PMC10295939; doi:10.3390/bios13060578)
Supplement: Supplementary file 1 [file biosensors-13-00578-s001.zip › biosensors-2269877-supplementary.pdf]

Supplementary information

Electrochemical sensing of dopamine using polypyrrole/Molybdenum Oxide modified ITO electrode

Nadiyah Alahmadi [1] and Waleed Ahmed El-Said [1,2] *

[1] Department of Chemistry, College of Science, University of Jeddah, Jeddah 21959, Saudi Arabia
[2] Chemistry Department, Faculty of Science, Assiut University, Assiut 71516, Egypt

* Correspondence: awaleedahmed@yahoo.com

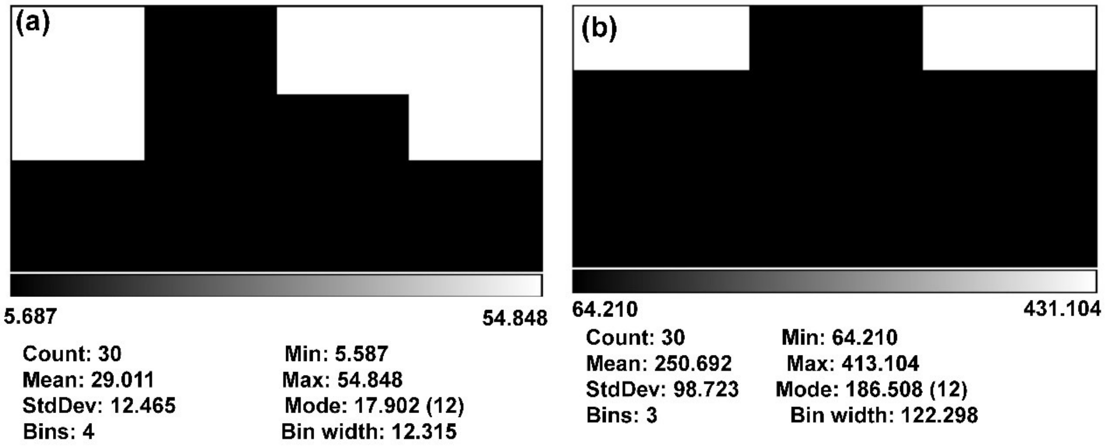

Figure S1. Distribution of the particle sizes of (a) MoO_3 NPs/ITO, and (b) PPy/MoO_3 NPs/ITO.
